# Biases in the measurement of health-related quality of life in the EQ-5D-5L among those with serious physical traumatic injuries: the ADVANCE cohort study

**DOI:** 10.1007/s11136-025-04158-9

**Published:** 2026-03-01

**Authors:** Daniel Dyball, Susie Schofield, Howard Burdett, Pete LeFeuvre, Alexander N. Bennett, Paul Cullinan, Christopher J. Boos, Anthony M. J. Bull, Nicola T. Fear

**Affiliations:** 1 King’s Centre for Military Health Research, King’s College London, Weston Education Centre, Cutcombe Road, London, SE5 9RJ UK; 2 Faculty of Medicine, Imperial College London, National Heart and Lung Institute, London, SW3 6LR UK; 3 Defence Healthcare Recovery Group, DMS Whittington, Lichfield, WS14 0FL UK; 4 Academic Department of Military Rehabilitation, Defence Medical Rehabilitation Centre, Stanford Hall Estate, Near Loughborough, Nottinghamshire, LE12 5BL UK; 5 London, UK; 6 Faculty of Health & Social Sciences, Bournemouth University, Bournemouth, BH1 3LT UK; 7 Department of Bioengineering, Centre for Blast Injury Studies, Imperial College London, London, SW7 2AZ UK; 8 Academic Department of Military Mental Health, King’s College London, London, SE5 9RJ UK

**Keywords:** Afghanistan, Military personnel, Wounds and injuries, Quality-adjusted life years, Quality of life, Bias

## Abstract

**Purpose:**

Physical traumatic injuries are likely to impact on a person’s Health-Related Quality of Life (HRQOL). We present data on HRQOL, from which EQ-5D-5L index scores are derived, and overall perceived health in UK Armed Forces personnel who sustained serious physical combat injuries and compare them to demographically similar personnel without such injuries (uninjured group). To ensure that perception of HRQOL is similar between groups, we also assess for measurement bias through Differential Item Functioning (DIF).

**Methods:**

577 personnel in an injured group (including subgroups of limb amputation injuries (n = 160) and non-amputation injuries (n = 417)) and 564 personnel in the uninjured group who sustained no such injuries were assessed in the ADVANCE cohort study. Health index values were derived from the EQ-5D-5L, overall perceived health from the EQ-visual analogue scale, and mobility from a Six-Minute Walk Test (6MWT). DIF was identified using multiple indicator multiple causes modelling.

**Results:**

Compared to the uninjured group, personnel injured with associated limb loss had significantly higher likelihood of reporting low health index scores (Relative Risk Ratio (RRR) 7.18 (95% Confidence Interval (CI) 4.25, 12.29; relative to high health index scores)), however no difference in the probability of reporting low or moderate overall perceived health, relative to high, was observed. Those injured without limb loss had a significantly higher probability of reporting low health index values (RRR 3.58 (95%CI 2.57, 5.03)) and low overall perceived health (RRR 1.91 (95%CI 1.34, 2.66)). Measurement bias was observed in items regarding mobility and anxiety/depression for those injured with associated limb loss and self-care for those with injured without. Differences in 6MWT only partially explained the bias observed in the mobility item.

**Conclusion:**

Personnel who experience serious traumatic injuries perceive aspects of HRQOL differently to personnel who experience no such injuries, with heterogenous biases expressed depending on presence of limb amputation. Researchers evaluating HRQOL in health interventions may need to account for this bias if comparing individuals with different traumatic injuries/conditions.

**Supplementary Information:**

The online version contains supplementary material available at 10.1007/s11136-025-04158-9.

## Plain language summary

Physical traumatic injuries have the potential to impact an individual’s quality of life. However, quality of life remains difficult to measure. If we ask a person before and after sustaining a physical traumatic injury about their quality of life, would we expect their quality of life to remain the same? Would we expect their idea of what quality of life is to also remain the same, or to change depending on the nature of their injury? In this study, we explore the health-related quality of life among UK Armed Forces personnel who sustained serious physical traumatic injuries and a comparison group of individuals who were similar to the injured group, but didn’t experience injuries. We find that whilst the injured group report worse health-related quality of life overall, we also observed some differences in how individuals perceive quality of life in terms of mobility, self-care and mental health that depended on the type of injury they received. Findings from this study show how researchers, policy makers and organisations must consider how to measure quality of life accurately, especially when comparing different groups of people.

**Supplementary Information:**

The online version contains supplementary material available at 10.1007/s11136-025-04158-9.

## Background

Health has been defined by the World Health Organization (WHO) as “a state of complete physical, mental and social well-being, and not merely the absence of disease and infirmity” [1]. The EuroQol 5-Dimension 5-Level (EQ-5D-5L) is one of the most widely used and validated measures of Health-Related Quality Of Life (HRQOL) worldwide [2–4]. The measure assesses HRQOL across dimensions of mobility, self-care, usual activities, pain/discomfort and anxiety/depression symptoms. Index values are derived from United Kingdom (UK) value sets undertaken by the general population [5]. The EQ-5D-5L has been recommended in the UK NHS as a patient-reported outcome measure to act as an indicator of HRQOL/successful outcome following major trauma and to improve services [6].

Physical traumatic injuries impact mental and physical health and well-being [7]. Specific types of injury, such as those with associated limb amputation, tend to be accompanied by a reduced ability to perform day to day activities, reduced mobility and overall lower HRQOL [8, 9]. Long-term impairment in HRQOL has been observed in both physical and emotional dimensions in seriously injured individuals, however it is noted that data on long-term health outcomes in trauma patients is lacking [10] and considerable heterogeneity in HRQOL outcomes exist depending on the nature of injury [11–13]. UK military personnel who sustained serious physical combat injuries in Afghanistan have greater odds of reporting probable mental health disorders and moderate to severe pain compared to demographically similar individuals who deployed but did not sustain a serious physical combat injury (uninjured group) [14, 15]. However, type of injury was important to consider, as those who experienced injuries with associated limb loss (amputation injury subgroup) had similar odds of reporting probable mental health disorders and moderate to severe pain compared to the uninjured group, whereas those who experienced injuries without associated limb loss (non-amputation injury subgroup) had increased odds of reporting these outcomes.

To accurately portray levels of HRQOL and other health outcomes, it is vital that questionnaires and surveys avoid bias and attain measurement invariance across the intended population/s [16]. Measurement invariance refers to the assumption that all items in a questionnaire measure the same underlying construct and are interpreted the same way by all those who complete it. One way to measure bias is through Differential Item Functioning (DIF), which occurs when different groups with the same level of an underlying latent trait (e.g. HRQOL) have dissimilar probabilities of reporting a symptom or health characteristic indicative of that trait [17]. For example, if a questionnaire intends to measure chronic pain, it may include items such as ‘pain in your knee’ or ‘pain in your foot’. If a person has a lower limb amputation and does not have a knee or foot, then they may score lower on this questionnaire, despite their actual levels of chronic pain. Thus, these questions have introduced bias towards this group. A measure is DIF-free if all groups have similar probabilities of endorsing a symptom at all levels of the latent trait (e.g. low, moderate and high HRQOL). When the probability of endorsing a symptom in one group compared to another is consistently higher across all levels of the latent trait, this is called uniform-DIF. The presence of DIF is problematic, as it represents biases that may over- or under-estimate actual levels of health domains within certain groups, making it difficult or impossible to compare groups effectively. Recent literature has highlighted the importance of not only identifying DIF, but also attempting to explain why DIF might exist [17]. This can be done through Multiple Indicator Multiple Causes (MIMIC) modelling, whereby potential reasons for DIF can be added as a mediator of the relationship between the exposure and item with identified DIF [18]. DIF has been identified in the EQ-5D-5L previously amongst patients following different surgical procedures [19].

### Aims

This study aims to a) compare HRQOL data from the EQ-5D-5L for a group of male UK Armed Forces personnel who sustained serious physical combat injuries in Afghanistan to a demographically similar group who sustained no such injuries by assessing differences in index values and self-reported overall health, b) assess whether DIF exists between these groups in the EQ-5D-5L, and c) attempt to explain observed DIF through the use of mediated-MIMIC modelling.

## Methods

### Participants

Participants took part in the ADVANCE cohort study, a study designed to assess the long-term physical and psychosocial functioning of UK Armed Forces personnel who sustained serious physical traumatic combat injuries in Afghanistan [20]. In brief, a group of male injured personnel who required aero-medical evacuation from Afghanistan to a UK hospital were identified by the UK Ministry of Defence (Defence Statistics). An additional group of male personnel were identified who deployed but did not sustain such injuries were frequency-matched to the injured group based on demographic characteristics such as age, rank, role on deployment, regiment, and specific deployment era. This paper uses the baseline assessment data of the ADVANCE cohort (data collected between 2015 and 2020) (n = 1145).

### Procedure

Participants attended the Defence Medical Rehabilitation Centre at Headley Court (2015–2018) or Stanford Hall (2018–2020). Participants completed a comprehensive suite of investigations, of which this analysis utilises the research nurse-led clinical interview, a six-minute walk test and self-report questionnaire.

## Materials

### Outcomes

#### Health-related quality of life (HRQOL)

HRQOL was assessed using the EUROQOL 5 Dimensional 5 Level (EQ-5D-5L) questionnaire [4]. This measure includes five items addressing problems with; mobility, self-care, usual activities, pain/discomfort and anxiety/depression symptoms. Items were coded from 1 (e.g. I have no problems in walking about) to 5 (e.g. I am unable to walk about).

#### Health index values

Index values were derived from the EQ-5D-5L and are scores between − 0.594 and 1, where 1 indicates perfect health, 0 indicates a health state equivalent to being dead and negative values indicate health states considered worse than death. Index values were generated using cross-walk procedures based on UK value sets [21]. Responses were categorised into tertiles of the entire cohort as poor (− 0.383–0.767) moderate (0.768–0.877) and high health index values (0.879–1.000).

#### Overall perceived health

The EQ-5D-5L also includes a component on overall perceived health called the EUROQOL-Visual Analogue Scale (EQ-VAS) [4]. Participants are asked to rate their health today from 0 (worse health imaginable) to 100 (best health imaginable). Responses were categorised into tertiles of the entire cohort as low (0–74) moderate (75–87) and high (88–100) perceived health.

#### Independent variables

### Six-minute walk test (6MWT)

A 6MWT was included as part of the baseline assessment [22], involving participants continuously walking during a six-minute period. Distance covered was measured in total metres travelled. Those with lower-limb amputation injuries completed the 6MWT with prosthetics. Participants who did not have access to prosthetics on the assessment day did not complete the 6MWT.

### Combat injury

Combat injury was established from Defence medical records, information provided by the Ministry of Defence (Defence Statistics) and supplemented by participants self-reports during the research nurse-led clinical interview. Amputation injuries were defined as injuries with associated limb loss (e.g. transhumeral, transtibial). Non-amputation injuries were defined as injuries without associated limb loss, details of which can be found in Dyball et al. 2022 [14]. Isolated partial amputation (e.g. digit, toe, partial foot) were not included in the amputation injury group unless accompanied by major limb amputation.

### Data analysis

The statistical software package STATA version 18.0 was used for data analysis. Due to a very small number of participants indicating the last response to each EQ-5D-5L item (e.g. for the mobility item, “I am unable to walk about”), response four and five were merged to allow models to converge. Known differences in pain and mental health outcomes between those who sustained amputation injuries and non-amputation injuries [14, 15] led to these groups being assessed separately. All models were bootstrapped utilising 1000 replications and bias-corrected confidence intervals are presented.

### Health index values/overall perceived health

A multinomial logistic regression model was utilised to assess whether sustaining serious physical combat injury was associated with health index values and overall perceived health, including assessment of injury subgroups (amputation and non-amputation). This model controlled for socioeconomic status (rank at sampling) and age at assessment [23, 24]. Relative Risk Ratios (RRR) and 95% confidence intervals are reported.

### DIF: MIMIC model

To assess DIF, MIMIC modelling was conducted using generalised structural equation modelling [25]. EQ-5D-5L items were modelled using ordinal family and probit links. As recommended by Euroqol, DIF was assessed using the raw EQ-5D-5L scores, not the index values scores [26]. Amputation and non-amputation injuries were dummy coded and compared to those who sustained no such injuries. An iterative scale purification approach to assess best anchoring items was used [25]. Each item was assessed for uniform DIF, with all other items constrained. Constrained items act as anchors/measures of the latent construct, in this case HRQOL, that are free of contamination e.g. no DIF. This process was repeated for each item. Items with significant DIF, as defined by a significant direct effect (*p* < .05), were then unconstrained, and the procedure repeated. The process ended once the same set of items are identified as having DIF in two consecutive iterations.

### DIF: mediated MIMIC model

**Fig. 1:**
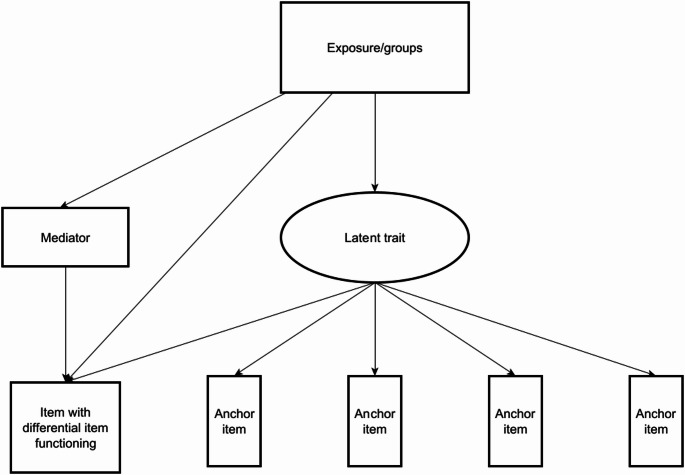
Example multiple indicator multiple cause model assessing differential item functioning with mediator

To explain potential causes of DIF, a mediated MIMIC model was performed [18]. Mediation models allowed all items with identified uniform DIF to vary and used all other items as anchors for the HRQOL latent trait (Fig. 1). A single mediator was introduced for item(s) exhibiting DIF. The product of coefficient method was used to assess the direct effect, the effect of exposure on the item not through the mediator, and indirect effect, the effect of exposure on the item through the mediator. Proportion mediated was estimated by the equation $\frac{\text{direct effect}*(\text{indirect effect}-1)}{(\text{direct effect}*\text{indirect effect})-1}$. The 6MWT was rescaled (divided by 100) for this model to increase statistical interpretability.

### Missing data/exclusions

One participant was excluded from these analyses as a result of experiencing serious injury outside of military service. 23 participants had missing data on the EQ-5D-5L, ranging from one item (n = 18), two items (n = 2) and all five items (n = 3). Participants with all five items missing were excluded from this analysis. Data was imputed using twoway imputation of standard errors for those with only one/two missing items [27, 28]. Due to the low level of missingness, casewise deletion was used to handle all other missing data. Some participants were unable to complete the 6MWT (n = 34), these participants were excluded from the mediated MIMIC model for mobility only. Additionally, participants who were < 25 (n = 6) or ≥ 50 (n = 10) years old at time of assessment and participants who walked over 1000 m on the 6MWT (n = 1) were excluded from the mediated MIMIC model for mobility due to problems with convergence when included, leaving a total of 1088 participants in the sample for the mediated MIMIC model.

## Results

1141 out of 1145 participants were included in this analysis (99.5% of the total study sample), 577 of which experienced serious physical traumatic injuries (160 with associated limb loss and 417 injured without associated limb loss) and 564 who experienced no such injuries (uninjured group). The median age of the cohort at time of assessment was 33 years (Table 1). The majority of the sample were junior non-commissioned officers/other ranks (71.8%). Higher rates of moderate-extreme problems with mobility, self-care, usual activities, pain/discomfort and anxiety/depression symptoms were observed in the amputation and non-amputation injury subgroups compared to the uninjured group. Table 2 show the percentages of specific item responses to the EQ-5D-5L, stratified by injury status. The uninjured group reported a median health index score of 0.84 (Interquartile Range (IQR) 0.77, 1.00), the overall injured group 0.77 (0.68, 0.84), the amputation injury subgroup 0.77 (IQR 0.68, 0.84) and the non-amputation injury subgroup 0.77 (IQR 0.69, 0.85). The uninjured group reported a median EQ-VAS score of 80 (IQR 75, 90), the overall injured group 80 (IQR 65, 95), the amputation injury subgroup 85 (75, 90) and the non-amputation injury subgroup 80 (IQR 65, 90). Boxplots of health index scores and EQ-VAS scores can be found in supplementary materials 1.

**Table 1:** Demographic, mobility and EQ-5D-5L data for UK military personnel without injury (uninjured group), with injury (overall injured group) and subgroups of the injured group (amputation injury and non-amputation injury)

	Uninjured group N = 564	Overall injury group N = 577	Amputation injury subgroup N = 160	Non-amputation injury subgroup N = 417
Median age at sampled deployment/injury in years (IQR)	26 (23, 29)	25 (22, 29)	25 (22, 28)	25 (22, 29)
Median age at ADVANCE assessment in years (IQR)	34 (30, 37)	33 (30, 37)	32.5 (30, 36)	33 (30, 38)
Median number of years between sampled deployment/injury and ADVANCE assessment in years* (IQR)	8 (7, 9)	8 (7, 10)	7 (7, 9)	9 (7, 10)
Rank at sampled deployment/injury n (%)				
Junior non-commissioned officer/other rank	337 (66.3%)	412 (76.5%)	127 (84.0%)	284 (73.8%)
Senior non-commissioned officer	147 (24.8%)	106 (17.2%)	20 (11.0%)	86 (19.4%)
Officer	79 (8.9%)	59 (6.3%)	13 (5.0%)	46 (6.8%)
Amputation				
Upper limb amputation***	0 (0%)	16 (2.7%)	16 (10.3%)	0 (0%)
Lower limb amputation***	0 (0%)	156 (25.0%)	156 (97.4%)	0 (0%)
Six minute walk test distance travelled in metres, median (IQR)	630 (578, 691)	565 (472, 644)	490 (335, 555)	598 (514, 670)
EQ-5D-5L n (%)				
Moderate to extreme problems with mobility (% (95%CI))	17 (2.8% (1.7, 4.6)	100 (17.6% (14.6, 21.1))	41 (26.6% (20.1, 34.3))	59 (14.5% (11.3, 18.5))
Moderate to extreme problems with self-care (% (95%CI))	NR ~	NR ~	NR ~	13 (3.0% (1.7–5.2))
Moderate to extreme problems performing usual activities (% (95%CI))	31 (5.7% (4.0, 8.1))	75 (13.3% (10.7, 16.5))	18 (11.5% (7.3, 17.8))	57 (13.9% (10.8, 17.8))
Moderate to extreme discomfort/pain (% (95%CI))	63 (11.5% (9.0, 14.6))	140 (24.8% (21.3, 28.7))	31 (19.9% (14.2, 27.1))	109 (26.5% (22.3, 31.1))
Moderate to extreme anxiety/depression (% (95%CI))	55 (10.7% (8.3, 13.8))	99 (18.7% (15.6, 22.4))	19 (13.1% (8.4, 19.7))	80 (20.7% (16.9, 25.2))
EQ-5D-5L health index scores**				
Health index score median (IQR)	0.84 (0.77, 1.00)	0.77 (0.68, 0.84)	0.77 (0.68, 0.84)	0.77 (0.69, 0.85)
Health index score tertiles				
Low (-0.383–0.767)	128 (23.5% (20.0, 27.3))	264 (46.2% (41.9, 50.5))	87 (53.0% (44.9, 60.9))	177 (43.8% (38.8, 48.8))
Moderate (0.768–0.878)	176 (31.0% (27.2, 35.0))	180 (31.6% (27.8, 35.7))	47 (30.9% (23.9, 38.9))	133 (31.9% (27.4, 34.7))
High (0.879–1.000)	254 (45.5% (41.3, 49.8))	119 (22.2% (18.8, 26.0))	24 (16.1% (10.9, 23.1))	95 (24.3% (20.2, 29.0))
EQ-Visual Analogue Scale				
Overall perceived health median (IQR)	80 (75, 90)	80 (65, 90)	85 (75, 90)	80 (65, 90)
Overall perceived health tertiles				
Low (0–74)	136 (25.1% (21.5, 29.0))	196 (35.5% (31.6, 39.7))	39 (25.4% (19.0, 33.0))	157 (39.1% (34.3, 44.0))
Moderate (75–87)	225 (39.5% (35.4, 43.7))	200 (33.6%, 29.7, 37.6))	60 (35.1% (28.0, 43.0))	140 (33.0% (28.5, 37.8))
High (88–100)	198 (35.5% (31.5, 40.0))	178 (30.9% (27.2, 34.9))	61 (39.5% (31.9, 47.6))	117 (27.9% (23.7, 32.6))

Weighted percentages are presented alongside unweighted cell counts

*IQR* interquartile range, *NR* Not reported

^*^Calculated from the difference between age at index injury for injured personnel or age at sampled deployment + 0.5 (to reflect average age during sampled year) for the uninjured group and age at baseline/follow up assessment

^**^Generated from population norm reference data of the EQ-5D-5L from the UK general population

^***^Participants may have a combination of upper and lower limb amputations

~ Some data suppressed to allow for confidentiality in line with Defence Statistics rounding policy (https://www.gov.uk/government/publications/defence-statistics-policies/ministry-of-defence-disclosure-control-and-rounding-policy)

**Table 2:** Item level differences in EQ5D5L across injured groups and uninjured group

	Uninjured group (n = 564) N (%)	Amputation injury subgroup (n = 160) N (%)	Non-amputation injury subgroup (n = 417) N (%)
EQ1: mobility			
I have no problems walking about	490 (86.4)	62 (40.3)	282 (68.0)
I have slight problems walking about	58 (10.8)	57 (33.5)	76 (17.6)
I have moderate problems walking about	16 (2.8) ~	24 (15.4)	44 (10.5)
I have severe problems walking about/ I am unable to walk about	~	17 (10.8)	15 (3.9)
EQ2: self-care			
I have no problem washing or dressing myself	547 (96.8)	145 (91.2)	367 (87.6)
I have slight problems washing or dressing myself	17 (3.2) ~	13 (8.8) ~	37 (9.4)
I have moderate-problems washing or dressing myself	~	~	13 (3.0) ~
I have severe problems washing or dressing myself /I am unable to wash or dress myself	0 (0.0)	0 (0.0)	~
EQ3: Usual activities			
I have no problems doing my usual activities	461 (81.2)	108 (68.9)	262 (63.6)
I have slight problems doing my usual activities	72 (13.1)	34 (19.6)	98 (22.5)
I have moderate problems doing my usual activities	23 (4.4)	18 (11.5) ~	41 (9.8)
I am unable to do my usual activities	8 (1.3)	~	16 (4.1)
EQ4: pain/discomfort			
I have no pain or discomfort	276 (49.5)	45 (29.5)	111 (27.7)
I have slight pain or discomfort	226 (39.0)	84 (50.7)	195 (45.5)
I have moderate pain or discomfort	57 (10.4)	24 (15.3)	85 (19.9)
I have severe pain or discomfort/ I have extreme pain or discomfort	5 (1.1)	7 (4.6)	26 (6.9)
EQ5: anxiety/depression			
I am not anxious or depressed	380 (66.5)	109 (66.8)	235 (54.9)
I am slightly anxious or depressed	129 (22.9)	32 (20.2)	102 (24.7)
I am moderately anxious or depressed	38 (7.1)	10 (6.7)	50 (11.9)
I am severely anxious or depressed/ I am extremely anxious or depressed	17 (3.6)	9 (6.4)	30 (8.5)

Weighted percentages are presented alongside unweighted cell counts

~ Some data suppressed to allow for confidentiality in line with Defence Statistics rounding policy (https://www.gov.uk/government/publications/defence-statistics-policies/ministry-of-defence-disclosure-control-and-rounding-policy). Responses with very low cell counts have been merged with the prior response

Compared to the uninjured group, personnel who experienced injuries with associated limb loss had an elevated risk of reporting moderate (RRR 2.89 (95% Confidence Interval (CI) 1.70, 5.10)) or low health index values (RRR 7.18 (95%CI 4.25, 12.29)) relative to high (Table 3). However, the risk of reporting moderate or low overall perceived health relative to high was similar between the amputation injury subgroup (moderate: RRR 0.93 (95%CI 0.63, 1.37); low (RRR 0.92 (95%CI 0.57, 1.46)) and the uninjured group. Compared to the uninjured group, personnel injured without associated limb loss had an elevated risk of reporting moderate (RRR 2.00 (95%CI 1.45, 2.90)) and low health index values (RRR 3.58 (95%CI 2.57, 5.03)) relative to high, as well as low overall perceived health (RRR 1.91 (95%CI 1.34, 2.66)) relative to high. The risk of reporting moderate overall perceived health relative to high was similar between the non-amputation injury subgroup and uninjured group (RRR 1.07 (95%CI 0.78, 1.45)).

**Table 3:** Multinomial logistic regression model investigating EQ-5D-5L health index values and overall perceived health

	Health index value (EQ-5D-5L) relative risk ratio (95%CI*)		Overall perceived health (EQ-VAS) relative risk ratio (95%CI*)	
	Moderate (0.768–0.878) vs high (0.879–1.000)	Low (**− **0.383–0.767) vs high (0.879–1.000)	Moderate (75–87) vs high (88–100)	Low (0–74) vs high (88–100)
Uninjured group	Ref	Ref	Ref	Ref
Overall injury group	2.17 (1.55, 2.96)	4.28 (3.11, 5.84)	1.02 (0.77, 1.34)	1.58 (1.17, 2.12)
Amputation injury subgroup	2.89 (1.70, 5.10)	7.18 (4.25, 12.29)	0.93 (0.63, 1.37)	0.92 (0.57, 1.46)
Non-amputation injury subgroup	2.00 (1.45, 2.90)	3.58 (2.57, 5.03)	1.07 (0.78, 1.45)	1.91 (1.34, 2.66)

^*^bias-corrected bootstrapped confidence intervals

The results of the MIMIC models can be found in Table 4. Following the iterative process for identifying DIF and anchor items (supplementary materials 2), uniform DIF was identified for the EQ1 mobility item and the EQ5 anxiety/depression item for those in the amputation injury subgroup. These individuals had a greater likelihood of endorsing problems with mobility/walking about and a lower likelihood of endorsing problems with anxiety/depression compared to the uninjured group at all levels of HRQOL. Personnel in the non-amputation injury subgroup exhibited DIF for the EQ3 usual activities item. These individuals had a lower likelihood of endorsing problems with usual activities item compared to the uninjured group at all levels of HRQOL.

**Table 4:** MIMIC model with unstandardised coefficients, adjusted for socioeconomic status at sampling and age at assessment

	EQ1-mobility coefficient (95%CI*)	EQ2-self-care	EQ3-Usual activities coefficient (95%CI*)	EQ4-pain/discomfort	EQ5-anxety/depression coefficient (95%CI*)
MIMIC model					
Amputation injury					
Direct effect	1.50 (1.1 7, 1.83)	0.38 (− 0.15, 0.94)	**− **0.10 (**− **0.48, 0.32)	0.08 (**− **0.38, 0.55)	− 0.28 **(−** 0.56, − 0.01)
Non-Amputation injury					
Direct effect	0.18 (**− **0.11, 0.42)	0.11 (**− **0.32, 0.48)	− 0.54 (− 1.05, − 0.16)	0.08 (**− **0.26, 0.24)	**− **0.09 (**− **0.28. 0.08)
Mediated MIMIC model					
Amputation injury					
Direct effect (Amputation injury- > EQ1)	0.82 (0.58, 1.26)	–	–	–	–
Indirect effect (Amputation injury- > distance travelled in six minute walk test- > EQ1)	0.50 (0.33, 0.70)	–	**–**	–	–
Proportion mediated $\frac{\text{direct effect}*(\text{indirect effect}-1)}{(\text{direct effect}*\text{indirect effect})-1}$	69%	**–**	–	–	–

^*^bias-corrected bootstrapped confidence intervals

**Fig. 2:**
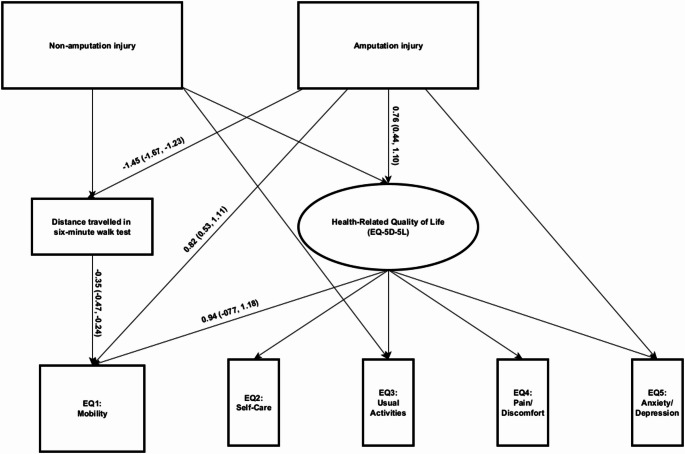
Mediated MIMIC model investigating differential item functioning in item one of the EQ-5D-5L mediated by distance travelled in the six-minute walk test (unstandardised coefficients and bias-corrected 95% confidence intervals)

A mediated-MIMIC model was created utilising the 6MWT as an objective indicator of mobility (Fig. 2). A significant indirect effect was observed between sustaining an injury with associated limb loss and the EQ-5D-5L item on mobility through the 6MWT, which explained 69% of the effect. The direct effect remained significant in this model, indicating that mobility as measured by distance travelled on the 6MWT only partially mediated the relationship.

## Discussion

In this analysis, we aimed to compare data from the EQ-5D-5L between UK Armed Forces personnel who sustained serious physical traumatic injuries and demographically similar personnel who sustained no such injuries and to assess whether there was any evidence of bias in measurement of HRQOL through DIF. We found that the injured group had significantly lower index values compared to the uninjured group. Despite this, those who sustained injuries with associated limb loss were just as likely to perceive their overall health as moderate to high as the uninjured group, whereas those who sustained injuries without associated limb loss had increased risk of reporting low overall perceived health. Bias in the measurement of HRQOL was observed through DIF in the EQ-5D-5L, with different injury types being associated with different measurement biases. Personnel in the amputation injury subgroup were more likely to endorse problems with mobility and less likely to endorse problems with anxiety/depression the EQ-5D-5L at all levels of HRQOL compared to the uninjured group. Differences in objective mobility as measured by distance travelled during the 6MWT only partially mediated the relationship between limb loss and responses on the mobility item. Personnel in the non-amputation injury subgroup were less likely to endorse problems with engaging in usual activities in the EQ-5D-5L at all levels of HRQOL compared to the uninjured group.

Whilst those who sustained injuries with associated limb loss were more likely to report lower health index values, their overall perceived health was not significantly different from the uninjured group. The odds of reporting probable mental illness including generalised anxiety, depression and PTSD have also been found to not significantly differ between these groups [14]. The amputation injury subgroup were also more likely to report post-traumatic growth, a positive psychological phenomena whereby individuals perceive beneficial psychological change following exposure to a trauma [29]. It is possible that through this positive lens, those injured with limb loss perceive their overall health to be relatively good [30], and may contribute to why we observed bias in the anxiety/depression item of the EQ-5D-5L between these groups. Other psychosocial factors may also be associated with perceived quality of life, such as social support [31]. However, previous research has identified that no significant differences in perceived social support are present between the injured and uninjured groups in the ADVANCE cohort [32].

We also observed bias in the mobility item of the EQ-5D-5L, whereby at all levels of HRQOL, those who sustained injuries with associated limb loss were more likely to endorse problems with mobility compared to the uninjured group. The mobility item measures one specific aspect of mobility; problems in “walking about”, which those with lower limb amputation injuries naturally have greater difficulty with. Whilst accounting for objective differences in mobility (6MWT) explained 69% of this association, DIF was still present in the model, suggesting that there are additional reasons why bias exists between these groups. It is possible that those who experience limb loss reflect more on the question of ‘problems in walking about’ than those who have not sustained such injuries. Pain/discomfort whilst walking, physical symptoms such as stiffness, difficulty in terrain etc. may influence their perception of problems in walking about, even when their overall HRQOL is relatively high [33].

Finally, we observed bias in the usual activities item of the EQ-5D-5L for those who sustained injuries without associated limb loss compared to those who sustained no serious physical traumatic injuries. The usual activities item refers to having problems doing activities such as work, study, housework, family or leisure activities. At all levels of HRQOL, personnel in the non-amputation injury subgroup were less likely than the uninjured group to endorse problems with conducting usual activities. This may seem paradoxical due to the fact that these individuals had higher rates of these problems compared to the uninjured group (moderate-extreme problems with usual activities 13.9% compared to 5.7% respectively), however there are multiple reasons why this might occur. DIF analysis adjusts for the level of the latent trait (HRQOL), which as observed, is significantly worse in the non-amputation injury subgroup. It is possible that personnel injured without limb loss have experienced times when their ability to perform ‘usual activities’ was at a much-reduced level (e.g. immediately post injury). As such, this might influence their perception of ‘no problems’, ‘slight problems’, ‘moderate problems’ etc. with these activities. This is known as response shift, whereby the experience of a trauma recalibrates (changes a respondent’s internal standards of measurement), reprioritises (changes the respondents’ values) or reconceptualises (changes the respondents’ definition of the construct) their perception on a particular issue [34, 35]. Personnel who sustained no serious injuries might perceive a smaller issue in their ability to perform usual activities as having a greater impact, whereas those who experienced injuries without associated limb loss, remembering a time when their ability to perform such activities were heavily reduced, might not perceive smaller issues as being particularly problematic.

Perceptions of HRQOL have been shown to be sensitive to response shift in people with disability [34], and may go some way to explaining the findings of this analysis. In the immediate aftermath of their injury, their perception of their current HRQOL would likely have been low, as their frame of reference would be when they were able-bodied prior to injury. As personnel progress in their rehabilitation at the Defence Medical Rehabilitation Centre, the salient experience of their health immediately post-injury becomes a new reference point and again impacts on how they reflect on their HRQOL. Increases in ability to maintain and use prosthetics (if necessary), increased understanding of their injuries and health-promoting behaviours would further impact their perceptions. At a median of 8 years following their injury/deployment of interest, at which point they were assessed for ADVANCE, some individuals may feel they have good mastery of their health [36–38]. It is possible that the phenomena of response shift will continue as participants age and age-related conditions/symptoms become more common or physiological musculoskeletal reserve is lost. Continued observation of this cohort may reveal effects such as this over time.

This paper has clinical and research implications. Whilst being one of the most widely used measures of HRQOL worldwide, the EQ-5D-5L is subject to internal standards/values which may change following major events, such as traumatic injury [34]. Researchers who wish to evaluate improvements in HRQOL following health-related intervention or compare groups of individuals may need to account for potential bias if investigated individuals have heterogeneous injuries/health conditions. Measures of HRQOL must take into account both objective as well as subjective aspects of HRQOL. A greater understanding of HRQOL may be derived from qualitative work, identifying aspects of HRQOL unique to certain groups of interest (e.g. those who have experienced traumatic injuries with/without associated limb loss) [39, 40]. The use of clinical vignettes, whereby individuals are asked about a range of hypothetical HRQOL situations, holds promise [41], and may be useful in calibrating the EQ-5D-5L to enhance response consistency [42].

Strengths of this analysis include a large sample size and use of novel, robust statistical approaches to assess measurement bias. However, there are multiple limitations to this study as well. One major limitation of the current analysis is that the multinomial logistic regression models utilised to assess health index values and overall perceived health cannot account for the effects of DIF and so should be interpreted cautiously. Health index values were generated based on cross-walk procedures from data collected in 1997 and no more recent datasets are available to compare to, meaning societal preferences that weighted responses may be outdated. Participants who were unable to complete the 6MWT were excluded from the mediated mobility model. These individuals were predominantly wheelchair bound personnel with amputation injuries. The amputation injury subgroup included individuals with upper and lower limb amputations, and those with isolated partial amputations were not included in the amputation subgroup. The number of upper limb amputations (without accompanying lower limb amputation) and isolated partial amputations was low. These individuals, as well as wheelchair bound personnel, likely have a unique experience of mobility issues that could not be accounted for in this analysis. No assessment of non-uniform DIF was undertaken, as this was beyond the scope of the current analysis [17]. Anchoring items, a process of choosing which items are DIF-free and good indicators of the latent trait, is an important aspect of assessing DIF [43]. When only a small number of items are available for investigation into DIF, as is the case for the EQ-5D-5L, this can considerably impact whether DIF is observed and the nature of the DIF observed [25, 43]. As such, we cannot guarantee that the DIF observed is not an artefact of the anchor selection procedure.

## Conclusions

UK Armed Forces personnel who sustain serious physical traumatic injuries have increased risk of reporting low HRQOL. However, those injured with associated limb loss perceived their overall health equivalently to demographically similar personnel who sustained no such injuries, whereas those injured without associated limb loss perceived their health to be worse. Differences in perceptions of HRQOL likely contribute to observed measurement bias in the EQ5D5L between those with/without serious physical traumatic injuries.

## Supplementary Information

Below is the link to the electronic supplementary material.

Supplementary Material 1

## Data Availability

The datasets generated and/or analysed during the current study are not publicly available due to the sensitive nature of the data but are available from the corresponding author on reasonable request. For enquiries to access data or discuss potential collaborations, please visit our data discovery page https://www.advancestudydmrc.org.uk/data-discoverability/ or contact adv_data_team@imperial.ac.uk.

## Abbreviations

DIF: Differential item functioning
HRQOL: Health-related quality of life
RRR: Relative risk ratio
WHO: World health organisation
6MWT: 6 Minute walk test
