# Supplementary material for: Biases in the measurement of health-related quality of life in the EQ-5D-5L among those with serious physical traumatic injuries: the ADVANCE cohort study
Source: Qual Life Res. 2026 Mar 1;35(4):81. doi: 10.1007/s11136-025-04158-9 (PMC12950649; doi:10.1007/s11136-025-04158-9)
Supplement: Supplementary file 1 — Supplementary Material 1 [file 11136_2025_4158_MOESM1_ESM.docx]

Supplementary materials 1: EQ-5D-5L Health Index Scores


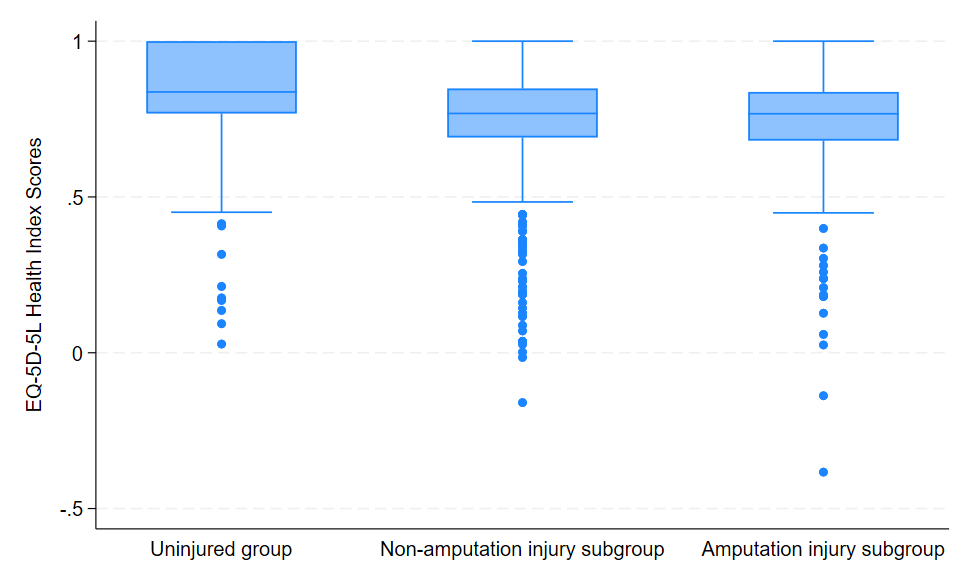


Supplementary materials 1: EQ-VAS Overall Perceived Health Scores


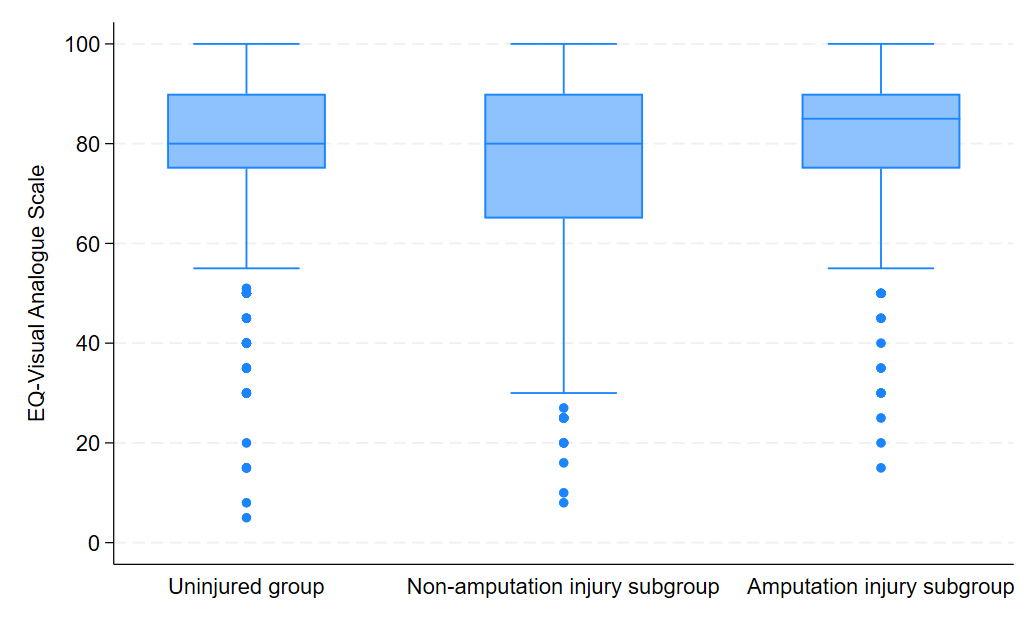


Supplementary materials 2: Anchor procedures for Differential Item Functioning

|  | EQ1 | EQ2 | EQ3 | EQ4 | EQ5 |
| --- | --- | --- | --- | --- | --- |
| Round1: all other items anchor |  |  |  |  |  |
| Amputation injury subgroup | **1.53 (1.24, 1.87)** | -0.12 (-0.73, 0.43) | **-0.95 (-1.63, -0.46)** | **-0.43 (-0.80, -0.07)** | **-0.44 (-0.71, -0.19)** |
| Non-amputation injury subgroup | **0.24 (0.01, 0.49)** | 0.16 (-0.26, 0.55) | **-0.55 (-1.12, -0.17)** | 0.03 (-0.23, 0.25) | -0.05 (-0.21, 0.11) |
| Round 2: all non-significant items anchor |  |  |  |  |  |
| Amputation injury subgroup | **1.33 (0.86, 1.87)** | - | -0.30 (-0.97, 0.43) | -0.11 (-0.56, 0.43) | **-0.28 (-0.60, -0.02)** |
| Non-amputation injury subgroup | 0.15 (-0.11, 0.41) | - | **-0.33 (-0.71, -0.00)** | -0.01 (-0.33, 0.28) | -0.07, -0.25, 0.10) |
| Round 3: all non-significant items anchor |  |  |  |  |  |
| Amputation injury subgroup | **1.52 (1.21, 1.90)** | 0.39 (-0.22, 0.98) | 0.01, -0.52, 0.49) | 0.39 (-0.03, 0.86) | **-0.28, -0.56, -0.03)** |
| Non-amputation injury subgroup | 0.18 (-0.08, 0.45) | 0.16 (-0.25, 0.59) | **-0.40 (-0.89, -0.02)** | 0.08 (-0.22, 0.38) | -0.07 (-0.24, 0.10) |
